# Gene Expression Associated with Early and Late Chronotypes in *Drosophila melanogaster*

**DOI:** 10.3389/fneur.2015.00100

**Published:** 2015-05-08

**Authors:** Mirko Pegoraro, Emma Picot, Celia N. Hansen, Charalambos P. Kyriacou, Ezio Rosato, Eran Tauber

**Affiliations:** ^1^Department of Genetics, University of Leicester, LeicesterUK

**Keywords:** chronotype, circadian clock, *Drosophila*, eclosion, transcriptomics

## Abstract

The circadian clock provides the temporal framework for rhythmic behavioral and metabolic functions. In the modern era of industrialization, work, and social pressures, clock function is jeopardized, and can result in adverse and chronic effects on health. Understanding circadian clock function, particularly individual variation in diurnal phase preference (chronotype), and the molecular mechanisms underlying such chronotypes may lead to interventions that could abrogate clock dysfunction and improve human (and animal) health and welfare. Our preliminary studies suggested that fruit-flies, like humans, can be classified as early rising “larks” or late rising “owls,” providing a convenient model system for these types of studies. We have identified strains of flies showing increased preference for morning emergence (Early or E) from the pupal case, or more pronounced preference for evening emergence (Late or L). We have sampled pupae the day before eclosion (fourth day after pupariation) at 4 h intervals in the E and L strains, and examined differences in gene expression by RNA-seq. We have identified differentially expressed transcripts between the E and L strains, which provide candidate genes for subsequent studies of *Drosophila* chronotypes and their human orthologs.

## Introduction

A broad range of life processes, physiological, biochemical, and behavioral, undergo regular daily rhythms that are driven by a circadian clock (1). In contrast to the period of these rhythms, which is highly uniform (24 h) among individuals, the timing (“phase”) of a given output of the clock can vary greatly. Humans, for example, show individual phase preference of activity and sleep, which allow one to be classified by a distinct chronotype, as either a “lark” or an “owl.” The study of chronotypes has been facilitated using a self-assessment questionnaire (2), which provided useful insights into different clock properties that are underlying diurnal preference. Furthermore, an accumulating body of evidence suggests that chronotypes have major impact in diverse areas from athletic performance (3) to personality traits underlying behavioral and emotional problems (4), risk taking propensity (5), and morality (6). Studies using monozygotic twins (7) and polymorphism in circadian clock genes (8) demonstrated a strong genetic basis for chronotype variation. However, the specific molecular mechanisms underlying diurnal preference are still obscure. Here, we have used *Drosophila melanogaster* as a model system for gaining an initial insight into the transcriptional changes associated with different chronotypes. The emergence of adult flies from their pupal case (eclosion) is an event that is tightly gated by the circadian system: it was the original phenotype used for screening for clock genes (9), and brain clock neurons (LNs) are required for correct gating (10). Importantly, while most flies eclose during dawn, a small proportion of flies often eclose at substantially delayed times (11). Furthermore, using artificial selection, it is possible to select for early and late eclosion chronotypes (12), indicating that there is a sizeable genetic component underlying variation in diurnal preference. Here, we have screened the *Drosophila* genetic reference panel, a suit of isogenic strains originated from the same wild population, whose complete genome has been sequenced (13). We have identified two strains that show robust early (E) and late (L) chronotypes and measured gene expression in these strains using RNA high-throughput sequencing during the 24 h before eclosion. To what extent does transcriptional variation associate with chronotype differences, and what are the associated differentially expressed genes (DEGs), are our two main research questions.

## Materials and Methods

### Eclosion measurement

For automatic monitoring of eclosion times, we developed an adaptor that fits the DAM2 system by TriKinetics (http://www.trikinetics.com). Our adaptor (called *Drosophila* eclosion logger adaptor; DELA) is made of Perspex, and the whole structure is placed in horizontal position, with modified (shortened) vertical activity tubes (Figure S1 in Supplementary Material). A single fly pupa was placed in each tube, just below the infra-red sensor of the DAM2. This design minimizes the time the fly needs to travel until detected by the infra-red sensor, and also takes advantage of the strong tendency of the fly to climb up (negative geotaxis). The advantage of this system compared to the Trikinetics eclosion monitor is that after the first crossing event detected by the sensor (and recorded by the computer), the fly is kept in the glass tube, rather than being drawn in a water–ethanol mixture. Flies can then be scored for gender or collected for further analysis or crossing. A custom made Perl script was used to extract the eclosion times from the TriKinetics data files.

### Sample collection and RNA extraction

A population of flies from each of the selected early and late line were set to lay eggs on apple juice media for 12 h. Newly hatched L1 instar larvae were selected from the media and moved to new vials to further synchronize individuals. The pupae were collected 1 day before eclosion at six time points as a mix of sexes. For LD samples, flies were kept in LD 12:12 throughout their entire development. For DD samples, flies were moved into constant darkness after 2 days of pupation. Total RNA was extracted from whole flies with Trizol. RNA-seq library preparation and sequencing was carried out by Beijing Genomics Institute BGI (Hong Kong, China). Following purification, the mRNA was fragmented using divalent cations at elevated temperature and the first-strand cDNA was synthesized using random hexamer primers and Superscript TM III (Invitrogen™, Carlsbad, CA, USA). The second strand cDNA was synthesized using buffer, dNTPs, RNaseH, and DNA polymerase I. Short fragments were purified with a QiaQuick PCR extraction kit (Qiagen) and resolved with EB buffer for end reparation and poly(A) addition. The short fragments were then connected using sequencing adapters. After agarose gel electrophoresis, suitable fragments were used as templates for PCR amplification. During the QC steps, an Agilent 2100 Bioanaylzer and an ABI StepOnePlus Real-Time PCR System were used in quantification and qualification of the sample library. Finally, the library (200 bp insert) was sequenced using Illumina HiSeq™ 2000 (Illumina Inc., San Diego, CA, USA). The single-end library was prepared following the protocol of the Illumina TruSeq RNA Sample Preparation Kit (Illumina).

### Sequence alignment

Single-end RNA-seq data were generated using Illumina HiSeq 2000 (BGI Tech Solutions, Hong Kong) and the adapter sequences were trimmed using trimmomatic (version 0.32) (14). Each library was quality checked using fastQC [version 0.11.2, (15)]. The total sequence obtained for each library ranged from 4.9 to 17.3 Mbp, with “per base quality score” >30 phred and “mean per sequence quality score” >33 phred. These RNA-seq reads were aligned to the *D. melanogaster* transcriptome (NCBI_build5.41) downloaded from the illumina igenome website. The libraries were alignment using TopHat (version 2.1.0) (16) with the following options: -i 50 -I 5000 – no-coverage-search – solexa1.3-quals -G genes.gtf (from NCBI_build5.41) as gene annotation reference. This procedure mapped between 67.9 and 84.7% of the total sequence, of which 3–4.2% resulted in multiple alignments. All the sequence reads of this study were deposited in the Sequence Reads Archive at the NCBI database under accession number SRP056783.

### Gene expression FPKM calculation

We used cufflinks and cuffmerge (version 2.2.1) (17, 18) to quantify the expression of the transcripts isoforms of the TopHat aligned RNA data. The options used were -u multi-reads correction, -g genes.gtf (from NCBI_build5.41) as gene annotation reference -b genome.fa (from NCBI_build5.41/Bowtie2Index) as reference genome -M Mask_sequences.fa (including mitochondrial and ribosomal RNA as well polyA, polyC sequences). The expression data were expressed in *Fragments Per Kilobase of transcript per Million fragments mapped* (FPKM).

### Gene expression analysis

To compare gene expression between E and L time-series samples, we employed the Time-series RNA-seq Analysis Package (TRAP) (19). To identify time-series DEGs, as well as relevant biological pathways, TRAP implements the over-representation analysis (ORA) (20), and pathway topology base analysis (SPIA) (21). TRAP combines pathway information from the public repository of pathway information Kyoto encyclopedia of gene and genomes – KEGG (22) with list of DEGs resulted by the time-series analysis to extrapolate differentially expressed pathways in a knowledge base-driven pathway analysis approach (23). TRAP identifies significant pathways by two methods: analysis of each time point (one-time point analysis) and summing across several time points (time-series analysis). The single time-point pathway analysis is initiated by identifying DEGs for each time point, which is followed by a single time-point ORA and SPIA to identify pathways that are significantly over represented among the DEGs. In the time-series pathway analysis, the “time” (and a time-lag factor) is considered as variable. This analysis returns a list of Time-series DEGs and Time-series pathways that are significantly represented among the Time-series DEGs.

### Network of interaction and pathway enrichment

Protein–protein interaction networks were built using Cytoscape Version 3.1.1(24). From a yeast 2-hybrid databases for *D. melanogaster* (BIOGRID-ORGANISM-Drosophila_melanogaster-3.2.105.mitab, including of 8210 nodes and 47383 interactions), sub-networks were isolated comprising the DEGs identify by the Time-series TRAP analysis and the proteins that interact with them (first neighbor nodes). To discover over-represented gene ontology (GO) categories of the genes in the sub-networks, we used the on-line tool DAVID, set at false discovery rates (FDR) <0.01, for the two estimates calculated by the program (25).

## Results and Discussion

**Figure 1:**
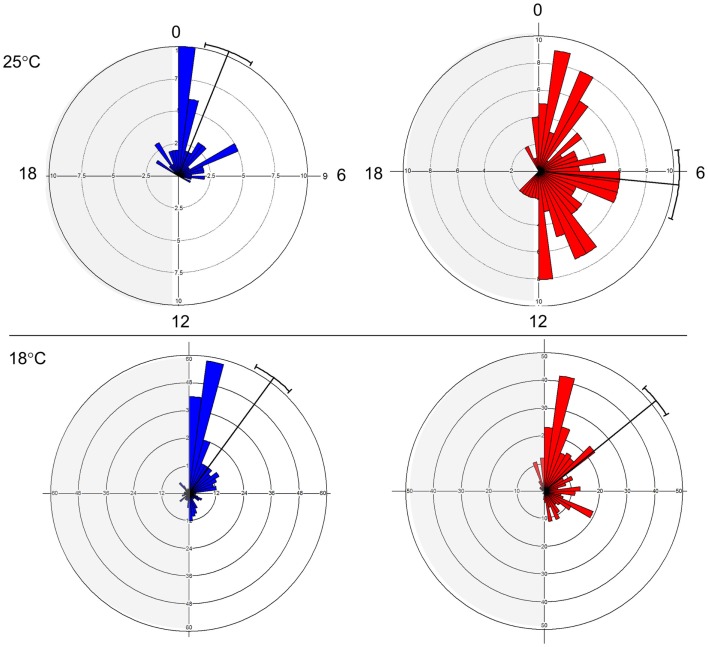
**Early and late eclosion chronotypes in *Drosophila***. Circular histograms showing eclosion times in E (blue; strain DGRP-371) and L (red; strain DGRP-385) chronotypes under LD condition. Plots show eclosion at 25°C (top) and 18°C (bottom). Mean eclosion vectors and 95% confidential limits are also depicted.

The E and L strains show a distinct phase of eclosion (Figure 1). At 25°C, E eclosed at Zt 1.47 ± 2.71 (mean vector ± SD), while L emerged at Zt 6.36 ± 4.79. The difference between the chronotypes was significant (Watson–Williams *F*_1,187_ = 55.78, *p* < 0.0001). At 18°C, the difference between the chronotypes became smaller, 2.41 ± 4.6 vs. 3.39 ± 4.13, but was still significant (*F*_1,702_ = 8.01, *p* < 0.05). Notably, there was a substantial increase in the phase distribution at the higher temperature, particularly in the L chronotype (Figure 1). This fits well with a previously proposed model (26), in which differences in the kinetics of accumulation of putative eclosion factors may affect phase synchrony among individuals. Under high temperature, when accumulation is rapid, more individuals are likely to reach the critical threshold and enter the eclosion gate, but would eclose at different phases within the gate. By contrast, under low temperature when the kinetic is slow, more individuals are likely to miss the gate and delay eclosion to the following day. Under this scenario, however, individuals will eclose at a relatively similar eclosion phase (e.g., synchronous). In addition, the difference in the way that the E and L responded to temperature (Figure 1) suggests that differences in both the threshold and the width of the eclosion gate contribute to the chronotype diversity.

**Figure 2:**
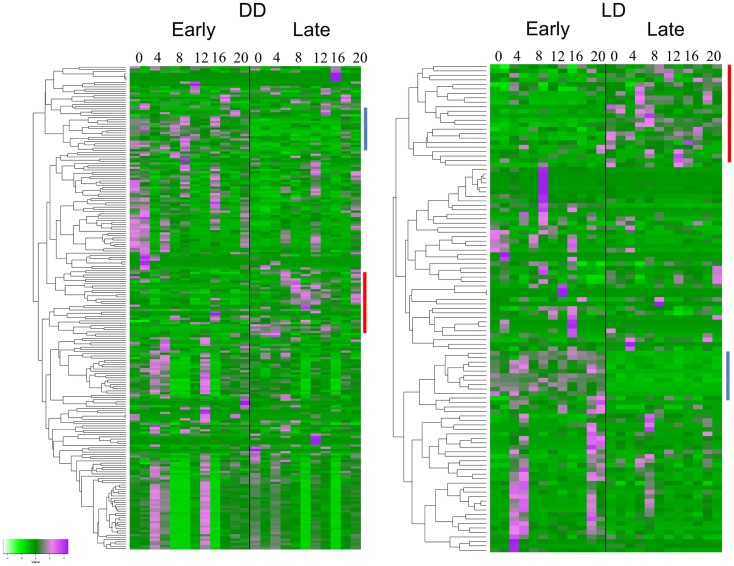
**Differentially expressed genes associated with eclosion chronotypes**. Hierarchical clustering of transcripts expression of genes identified by TRAP analysis. The heatmaps show the temporal expression profiles of the transcripts of 152 and 77 significant genes identified by time-series TRAP analysis for the DD experiment (left panel) and the LD experiment (right panel), respectively (with mean 0 and variance 1). The color scale (bottom left corner) illustrates the relative expression level across all samples: purple color represents an expression level above mean, bright green color represents expression lower than the mean. Each time point is represented by two independent replicates. The dendrograms on the left of each heatmap show the hierarchical clustering of the transcripts (for gene names, see Table S1 in Supplementary Material). Clusters of genes present in both DD and LD are indicated by lines with the same color.

The TRAP analysis revealed 152 genes that showed a significant different expression profile between E and L in DD, and 77 genes in LD (Figure 2). Sixty-four of the genes (83%) were present in both lists (Table S1 in Supplementary Material). Analysis of the heatmaps (Figure 2) clearly shows that difference between E and L is not merely a phase shift, as the expression pattern for a given transcript is entirely dissimilar between the two conditions. For example, a cluster of genes (including γ*Try*, *AdgfD*, and *CCKLR-17D3*, Figure 2) was activated in E, but silenced in L (both in DD and LD).

**Figure 3:**
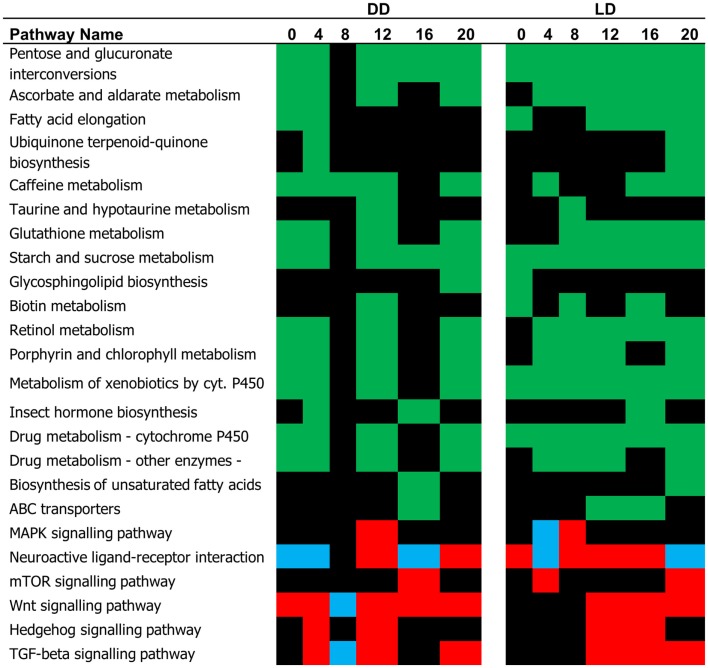
**Pathway analysis (single time point)**. The pathways identified by the TRAP algorithm based on significant gene expression at successive time points are shown. Based on the expression trend, these pathways can be predicted as being either activated (red, L relative to E) pG < 0.05, or inhibited (blue). The pathways whose status is undermined are depicted in green.

The difference in the expression profiles between E and L has been reflected in number of pathways that were significantly different between the two chronotypes (Figure 3). Many of these pathways were associated with metabolism. In some cases, where the DEG’s associated with a specific pathway showed a consistent trend of regulation, the TRAP algorithm could predict whether the pathway is activated or inhibited. For example, the mTOR, and Hedgehog signaling pathways have been predicted to be activated in L relative to E both in DD and LD (Figure 3). By contrast, the MAPK and the Wnt signaling pathways show both episodes of activation and inhibition at different time points.

**Figure 4:**
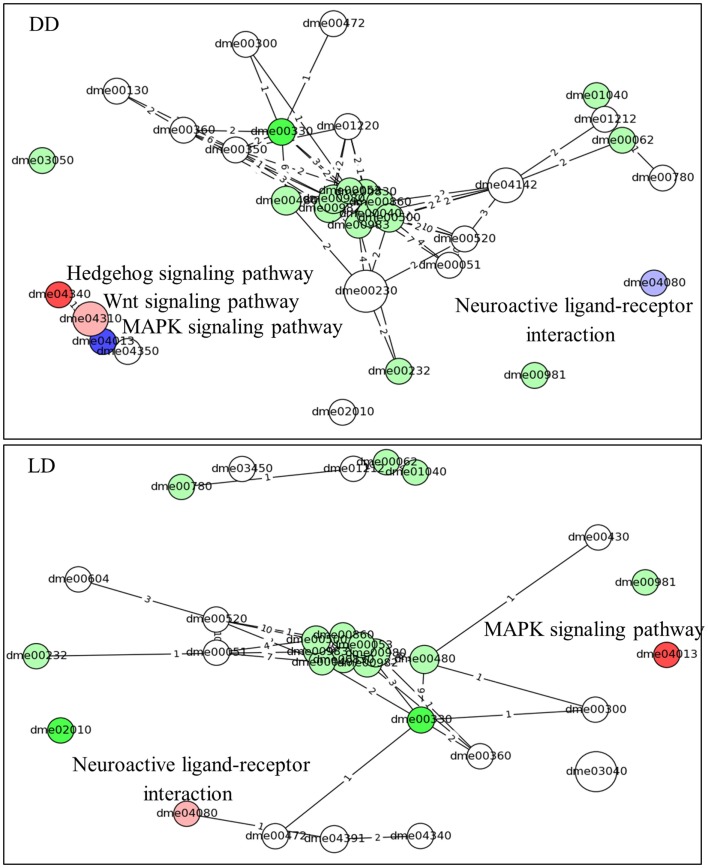
**Pathway analysis (time-series)**. The predicted pathways across the time series and their interactions are depicted. Nodes represent pathways (nodes size represents number of genes) and the edges signify shared genes between the nodes (no. of shared genes is shown). Nodes with large number of shared gene are draw near each other. The node’s color codes the status of the pathways (same as in Figure 3). The intensity of the nodes color is inversely proportional to pG values. The name of the pathways is coded accordingly to Kyoto encyclopedia of gene and genomes; KEGG (22). The pathways are listed in Table S2 in Supplementary Material.

Similar results are obtained using the time-series pathways analysis, where DEG’s expression is considered across the whole experiment (Figure 4; Table S2 in Supplementary Material). This analysis highlighted the Hedgehog signaling pathway as being activated in L compared to E (in DD, but not in LD). By contrast, the MAPK signaling and the Neuroactive ligand–receptor interaction (NLRI) pathways were strongly inhibited in DD, but intriguingly were activated in LD. As before, a substantial number of metabolic pathways were associated with the chronotypes and were highly interlinked, constituting a major network hub (Figure 4).

**Figure 5:**
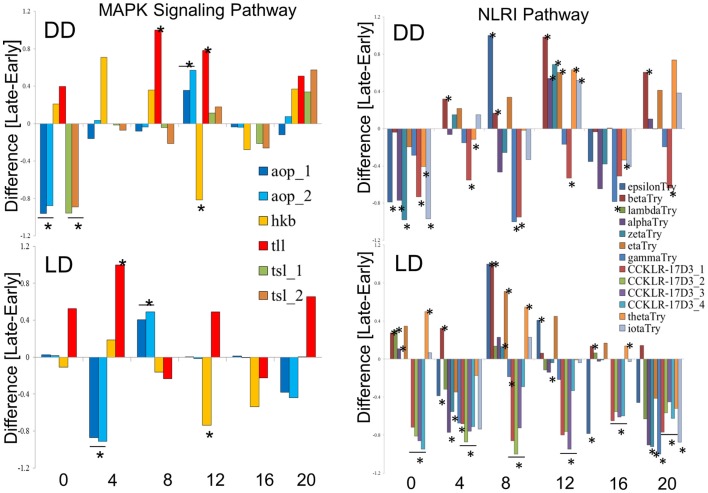
**Expression profiles of chronotype DEGs in DD and LD**. Difference between normalized FPKM values for the transcripts of DEGs identify by TRAP time-series analysis of the MAPK signaling pathway (left) and the Neuroactive ligand–receptor interaction pathway (NLRI, right). For each time point, significant difference is determined by single point TRAP analysis.

We explored in further details the expression of the DEGs in the MAPK and NLRI pathways (Figure 5). In the MAPK pathway, the *tailless* (*tll*), *huckebein* (*hkb*), and *anterior-open* (*aop*) genes showed similar profiles in DD and LD, while *torso-like* (*tsl*) differed between the chronotypes only in DD. We note that *tll* and *hkb* encode transcription factors, and all four genes are important for development. Of particular interest is *aop* (aka *yan*), which is involved in the development of the eye photoreceptor cells (27) and therefore may contribute to the underlying developmental differences between the chronotypes. In the NLRI, the profiles are less consistent between DD and LD. The cholecystokinin-like receptor 17D3 (*CCKLR-17D3*), encoding a G protein-coupled receptor (rhodopsin-like) differed only in LD (elevated in E compared to L). Members of the *Trypsin* (*Try*) cluster showed opposite trends in DD and LD.

Next, we have used the publically available protein–protein interaction data (28) to construct the protein network based on the chronotype DEGs (Figure S2 in Supplementary Material). The interactome associated with the chronotypes emerged as a sizable and highly connected network: in DD, 89 nodes were interacting with 618 proteins, resulting in 5057 edges. In LD, the network consisted of 45 nodes, connected to 363 other proteins (total of 3221 interactions).

**Figure 6:**
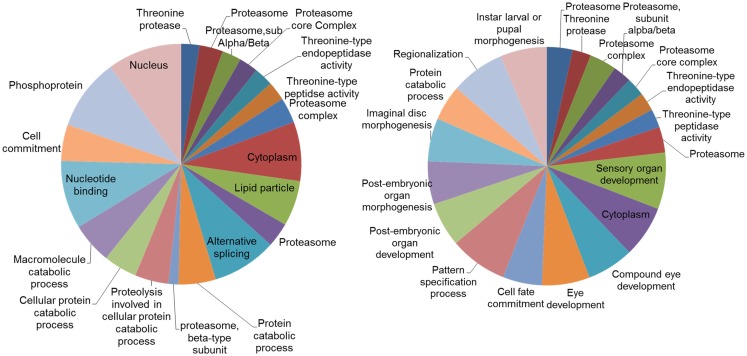
**Gene ontology (GO) categories of the genes identified by the networks analysis**. Pie charts of the 20 most significant enriched GO categories represented by the genes identified by the network analysis: DD on the left, LD on the right. The size of the section is proportional to the number DEGs in GO categories.

Analysis of the networks (DEGs and their first neighbors) for enriched gene ontologies revealed large number of significant GO terms (Figure 6). While part of these terms were common both in DD and LD, particularly terms associated with proteasome and threonine-type peptidase activity, distinct groups of GO terms were different. In LD, there were a number of GO terms associated with the post-embryonic and compound eye development; while in DD, there was enrichment of terms associated with alternative splicing and protein catabolic processes.

**Figure 7:**
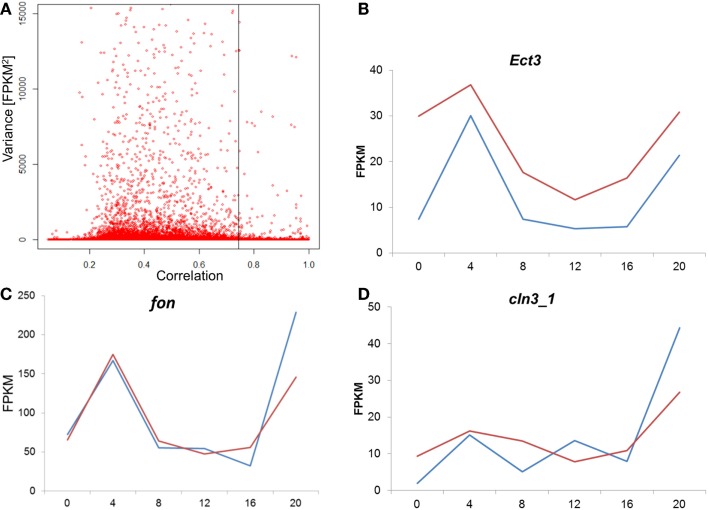
**Analysis of cross-correlation between E and L chronotypes**. **(A)** For each transcript, the cross-correlation between the E and L time-series was calculated and the maximal correlation score is plotted. As most of the transcripts that have constitutive expression (low variance) would show high correlation between E and L, we plot the data against the variance in reads number (FPKM). A permutated dataset was used to calculate the 5% FDR (shown as vertical line). In cases where correlation was high, the phase shift was minute as shown here in three examples **(B–D)**. Each point represents the average of two replicates libraries, for E (blue) and L (red). For more examples, see Figure S3 in the Supplementary Material.

To test whether the difference between the E and L chronotypes is simply driven by phase shift of gene expression, we tested the cross-correlation between the time series of the two chronotypes, for each transcript. In this analysis, the correlation between the two time-series is calculated repeatedly, at different time-lags. If the profiles are similar, but just phase shifted, the correlation will be at maximum at the lag that corresponds to the phase shift. Figure 7A shows the distribution of the maximal correlation scores for all the transcriptome. As most of the transcripts that have constitutive expression (low variance) would show high correlation between E and L, we plot the data against the variance in reads number (FPKM). At 5% FDR, the number of correlated transcripts between E and L is very small, particularly among those with high variance. In the few transcripts where correlation is present, the phase shift is minute, or non-existing (Figure 7; Figure S3 in Supplementary Material). Overall, this analysis indicates that the pattern of gene expression is substantially different between the chronotypes, and not merely phase shifted.

In general, our results suggest that chronotype diversity is largely mediated by genes, which are downstream of the circadian clock. None of the *Drosophila* core clock genes seems to show substantial expression difference between the E and the L chronotypes. Yet, it is possible that variation in clock genes drives different chronotypes post-transcriptionally. Indeed, previous studies demonstrated that variation in phase preference is often due to genetic variation in clock genes; for example, variation in the *per* gene between *D. melanogaster* and *D. pseudoobscura* are underlying phase differences in locomotor and sexual behavior rhythms (29). Another example is the common missense SNP, which has been recently identified in *Drosophila cry*, leading to variation in eclosion phase (30). Nevertheless, it seems that mutations that modify the output phase are more likely to occur in downstream targets rather than the clock itself, allowing phase variation in specific restricted functions without changing the global phase of the pacemaker.

To date, very little is known about the transcriptional variation between chronotypes in other model organisms. Our study may provide candidate genes and molecular pathways that could be explored in other insects and possibly even mammals, given the highly evolutionary conservation of the circuits that we identified here such as MAPK and Hedgehog. In addition, the possible link of chronotype variation to genes associated with development that we identified here, may well be relevant to mammalian systems that worth further investigation.

The general conclusion emerging from our time-series analysis is that gene expression is not merely phase shifted between the E and L chronotypes, but is more fundamentally affected (Figure 2). It seems that early differences in expression lead to different cascades in the different chronotypes, which is manifested, counterintuitively, by different enriched pathways leading to eclosion (Figure 4). This is reminiscent of Waddington’s concept of “epigenetic landscape,” where a single genotype can lead to different phenotypes (31), much like a ball that falls downhill along different valleys. In the case of eclososion chronotypes, however, the expression landscape consists of different valleys converging to a single outcome (eclosion). The gene expression program underlying chronotype diversity is fittingly illustrated by paraphrasing the old saying: all roads lead to Rome, but travel time may vary.

## Author Contributions

EP and CH carried the phenotypic analysis and collected the samples. MP assembled the sequence data and carried the analysis. ET, ER, and CK designed the experiments. ET and ER supervised the experiments. ET, MP, ER, and CK contributed to the preparation of the manuscript.

## Conflict of Interest Statement

The authors declare that the research was conducted in the absence of any commercial or financial relationships that could be construed as a potential conflict of interest.

## Supplementary Material

The Supplementary Material for this article can be found online at http://journal.frontiersin.org/article/10.3389/fneur.2015.00100/abstract
