# Supplementary material for: Gene Expression Associated with Early and Late Chronotypes in Drosophila melanogaster
Source: Front Neurol. 2015 May 8;6:100. doi: 10.3389/fneur.2015.00100 (PMC4457141; doi:10.3389/fneur.2015.00100)

Supplementary Material

Gene Expression Associated with Early and Late Chronotypes in *Drosophila melanogaster*

Pegoraro M, Picot E*, Hansen C*, Kyriacou CP, Rosato E, Tauber E[$]

Dept. Genetics, University of Leicester, Leicester United Kingdom

***** Equal contribution

[$] **Correspondence:** Dr Eran Tauber Dept. of Genetics. University of Leicester. Leicester LE1 7RH United Kingdom
et22@le.ac.uk

Fig. S1. **Drosophila Eclosion Logger Adaptor (DELA).** The adaptor fits the TriKinetics DAM2 monitor and the whole structure is placed in horizontal position, with modified (shortened) vertical activity tubes. A single fly pupa was placed in each tube, just below the infra-red sensor of the DAM2. This design minimises the time the fly needs to travel until detected by the infra-red sensor, and also takes advantage of the strong tendency of the fly to climb up (negative geotaxis).

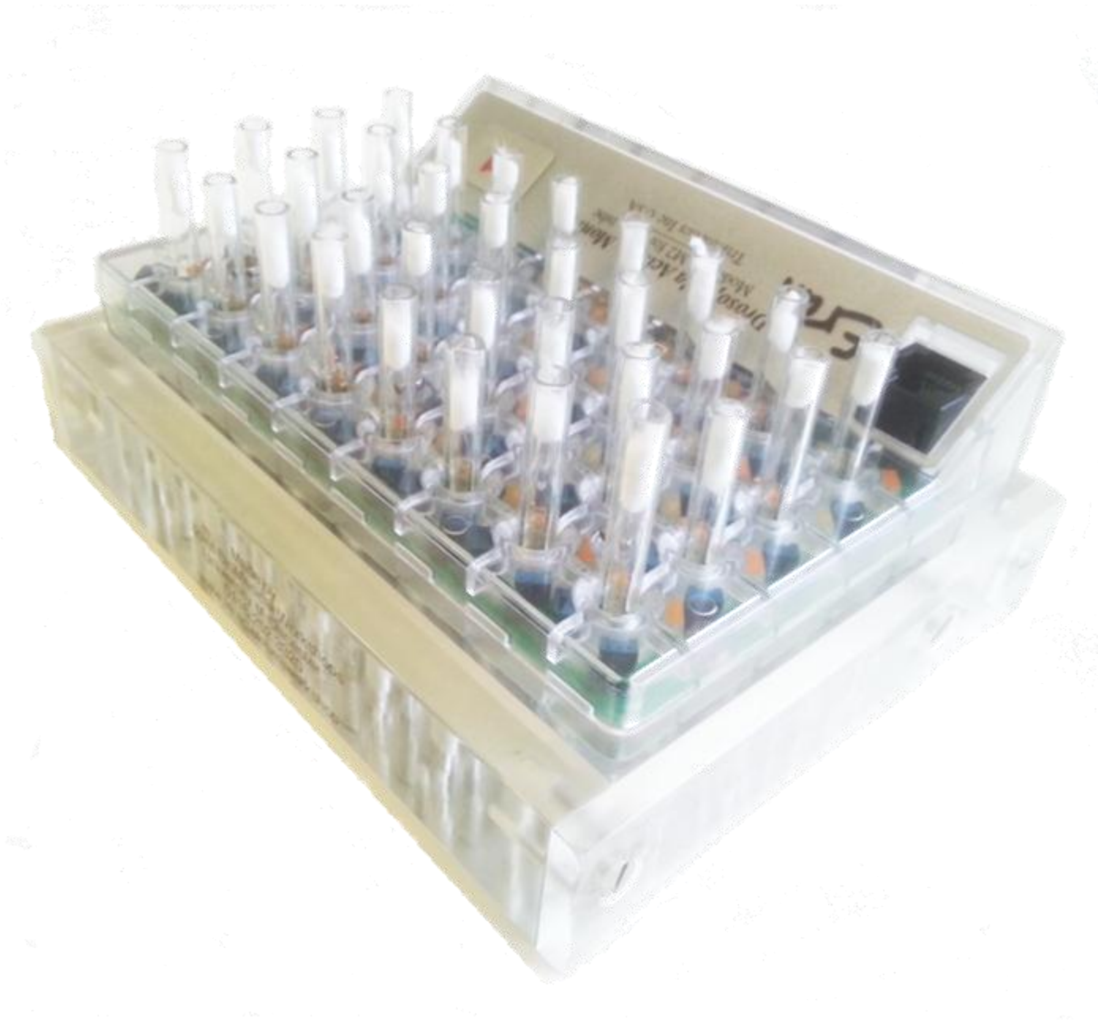

Supplement: Supplementary file 1 [file Image_1.PDF]
