# Supplementary material for: Gene Expression Associated with Early and Late Chronotypes in Drosophila melanogaster
Source: Front Neurol. 2015 May 8;6:100. doi: 10.3389/fneur.2015.00100 (PMC4457141; doi:10.3389/fneur.2015.00100)

Supplementary Material

Gene Expression Associated with Early and Late Chronotypes in *Drosophila melanogaster*

Pegoraro M, Picot E*, Hansen C*, Kyriacou CP, Rosato E, Tauber E[$]

Dept. Genetics, University of Leicester, Leicester United Kingdom

***** Equal contribution

[$] **Correspondence:** Dr Eran Tauber Dept. of Genetics. University of Leicester. Leicester LE1 7RH United Kingdom
et22@le.ac.uk

Fig. S2. Protein interaction networks. The networks were generated using the proteins encoded by the chronotype DEGs and their predicted first neighbour. The network for the DD experiment (left) is composed by 89 nodes from the time-series TRAP analysis interacting with 618 first neighbour proteins resulting in 5057 edges. The LD experiment (right) network contains 45 nodes interacting with 363 first neighbour nodes (3221 edges). Green nodes are DEGs from the time-series TRAP analysis unique for the DD or LD experiments. Red nodes are DEGs from the time-series TRAP analysis common between the two experiments.

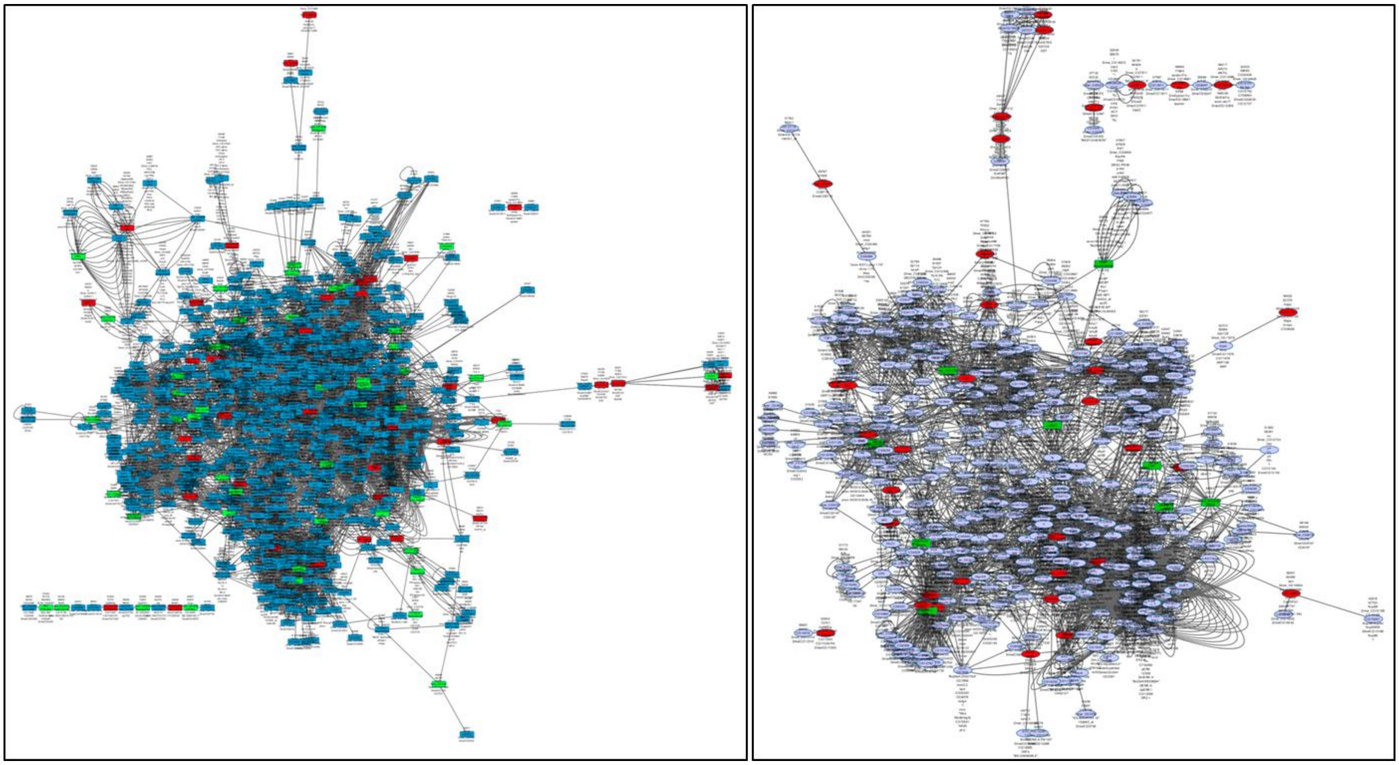

Supplement: Supplementary file 2 [file Image_2.PDF]
