# Supplementary material for: Gene Expression Associated with Early and Late Chronotypes in Drosophila melanogaster
Source: Front Neurol. 2015 May 8;6:100. doi: 10.3389/fneur.2015.00100 (PMC4457141; doi:10.3389/fneur.2015.00100)

Supplementary Material

Gene Expression Associated with Early and Late Chronotypes in *Drosophila melanogaster*

Pegoraro M, Picot E*, Hansen C*, Kyriacou CP, Rosato E, Tauber E[$]

Dept. Genetics, University of Leicester, Leicester United Kingdom

***** Equal contribution

[$] **Correspondence:** Dr Eran Tauber Dept. of Genetics. University of Leicester. Leicester LE1 7RH United Kingdom
et22@le.ac.uk

Fig. S3. Expression profiles in E and L chronotypes. Nine examples of high cross-correlation between E (blue) and L (red), showing similar phase. Each point represents the average of two replicates libraries.

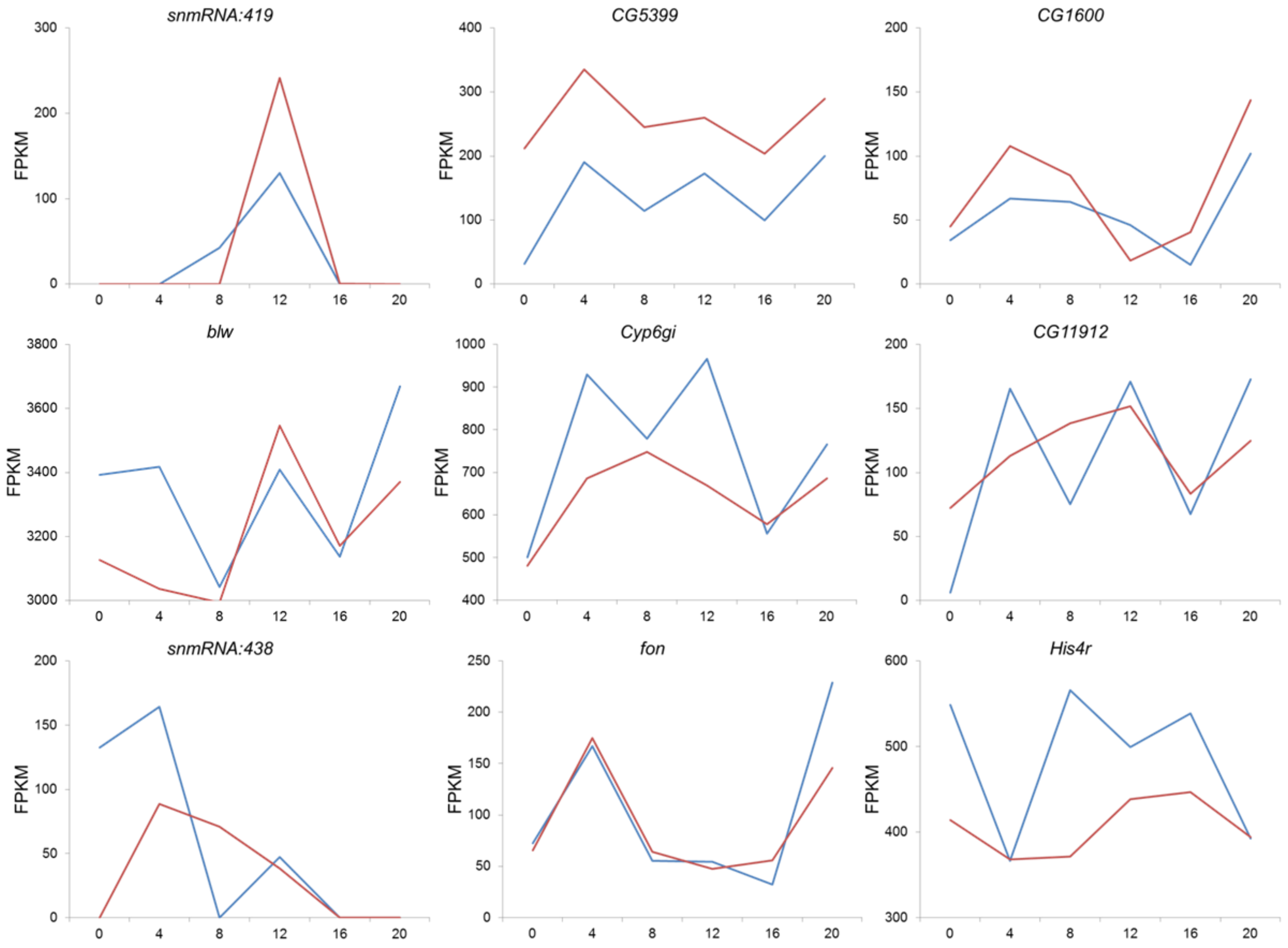

Supplement: Supplementary file 3 [file Image_3.PDF]
