# Supplementary material for: Gene Expression Associated with Early and Late Chronotypes in Drosophila melanogaster
Source: Front Neurol. 2015 May 8;6:100. doi: 10.3389/fneur.2015.00100 (PMC4457141; doi:10.3389/fneur.2015.00100)
Supplement: Supplementary file 4 [file Table_1.PDF]

Supplementary Material

Gene Expression Associated with Early and Late Chronotypes in *Drosophila melanogaster*

Pegoraro M, Picot E*, Hansen C*, Kyriacou CP, Rosato E, Tauber E[$]

Dept. Genetics, University of Leicester, Leicester United Kingdom

***** Equal contribution

[$] **Correspondence:** Dr Eran Tauber Dept. of Genetics. University of Leicester. Leicester LE1 7RH United Kingdom
et22@le.ac.uk

TableS1. Differentially expressed genes between E and L chronotypes.

DD		LD	
Transcript Name	Molecular Function	Transcript Name	Molecular Function
Gα49B	G-protein coupled receptor binding	*Gα49B*	G-protein coupled receptor binding
Gα49B	G-protein coupled receptor binding	*Gα49B*	G-protein coupled receptor binding
desat2	stearoyl-CoA 9-desaturase activity	*upd3*	positive regulation of JAK-STAT cascade
Gα49B	G-protein coupled receptor binding	*wntD*	G-protein coupled receptor binding
Gyc-89Da	guanylate cyclase activity	*Gα49B*	G-protein coupled receptor binding
Gα49B	G-protein coupled receptor binding	*Gα49B*	G-protein coupled receptor binding
Gyc-89Db	guanylate cyclase activity	*Ect3*	beta-galactosidase activity
CG9517	..	*Amy-d*	cation binding
kkv	chitin synthase activity	*Amy-p*	cation binding
Ugt37c1	glucuronosyltransferase activity	*Fad-2*	stearoyl-CoA 9-desaturase activity
GstD6	glutathione transferase activity	*PH4αNE1*	procollagen-proline 4-dioxygenase activity
Gα49B	G-protein coupled receptor binding	*βTry*	serine-type endopeptidase activity
spn-D	DNA-dependent ATPase activity	*Ugt86i*	glucuronosyltransferase activity
Ugt37b1	glucuronosyltransferase activity	*Takl2*	protein kinase activity
Gα49B	G-protein coupled receptor binding	*Wnt10*	G-protein coupled receptor binding
kkv	chitin synthase activity	*Sodh-2*	L-iditol 2-dehydrogenase activity
PH4αNE1	procollagen-proline 4-dioxygenase activity	*Mdr50*	drug transmembrane transporter activity
dib	iron ion binding	*spo*	iron ion binding
Adh	alcohol dehydrogenase (NAD) activity	*vri*	sequence-specific DNA binding
GstD7	glutathione transferase activity	*Ugt37c1*	glucuronosyltransferase activity
γTry	serine-type endopeptidase activity	*Gα49B*	G-protein coupled receptor binding
ade5	phosphoribosylaminoimidazolesuccinocarboxamide synthase activity	*CG9517*	..
Adgf-D	adenosine deaminase activity	*Hsp70Aa*	..
trpl	cation channel activity	*GstD5*	glutathione transferase activity
trpl	cation channel activity	*Hsp70Bc*	..
ade5	phosphoribosylaminoimidazolesuccinocarboxamide synthase activity	*Hsp70Bb*	..
CCKLR17D3	neuropeptide receptor activity	*Hsp70Bbb*	..
Prx2540-1	peroxidase activity	*GstD6*	glutathione transferase activity
Adh	alcohol dehydrogenase (NAD) activity	*Hsp70Ab*	..
GstD4	glutathione transferase activity	*GstD4*	glutathione transferase activity
proPO-A1	catechol oxidase activity	*upd2*	positive regulation of JAK-STAT cascade
Ugt86De	glucuronosyltransferase activity	*GstD2*	glutathione transferase activity

Ugt86Dd	glucuronosyltransferase activity
Cht8	chitin binding
GstD2	glutathione transferase activity
GstD5	glutathione transferase activity
Gpdh	glycerol-3-phosphate dehydrogenase [NAD+] activity
PH4αPV	procollagen-proline 4-dioxygenase activity
Gα49B	G-protein coupled receptor binding
Cht4	chitin binding
Uro	urate oxidase activity
Gα49B	G-protein coupled receptor binding
Ugt36Bb	glucuronosyltransferase activity
Sk1	D-erythro-sphingosine kinase activity
trpl	cation channel activity
Adh	alcohol dehydrogenase (NAD) activity
θTry	serine-type endopeptidase activity
αTub67C	structural constituent of cytoskeleton
Gα49B	G-protein coupled receptor binding
shf	protein binding
Ahcy89E	adenosylhomocysteinase activity
ple	tyrosine 3-monooxygenase activity
Adh	alcohol dehydrogenase (NAD) activity
ζTry	serine-type endopeptidase activity
iTry	serine-type endopeptidase activity
αTry	serine-type endopeptidase activity
Adh	alcohol dehydrogenase (NAD) activity
Ugt37a1	glucuronosyltransferase activity
GstE4	glutathione transferase activity
Ahcy89E	adenosylhomocysteinase activity
Tps1	alpha,alpha-trehalose-phosphate synthase (UDP-forming) activity
Aph-4	alkaline phosphatase activity
shf	protein binding
cln3	..
LKR	saccharopine dehydrogenase (NAD+, L-lysine-forming) activity
tsl	torso binding
jhamt	juvenile hormone acid methyltransferase activity
ple	tyrosine 3-monooxygenase activity
CG33138	cation binding
Hex-C	fructokinase activity
tsl	torso binding
wmd	receptor binding
Tsp42Eb	..
b	pyridoxal phosphate binding
Cht5	chitin binding
aop	sequence-specific DNA binding transcription factor activity
Gα49B	G-protein coupled receptor binding
Gα49B	G-protein coupled receptor binding
Gα49B	G-protein coupled receptor binding
Vkor	vitamin-K-epoxide reductase (warfarin-sensitive) activity
Gα49B	G-protein coupled receptor binding
Gα49B	G-protein coupled receptor binding
snRNA:U2:34ABa	..
proPO-A1	catechol oxidase activity
Gα49B	G-protein coupled receptor binding
Gα49B	G-protein coupled receptor binding
Gα49B	G-protein coupled receptor binding
Gα49B	G-protein coupled receptor binding
desat2	stearoyl-CoA 9-desaturase activity
βTub60D	GTPase activity
Cht3	chitin binding
Cht7	chitin binding
ds	cadherin binding
Gα49B	G-protein coupled receptor binding
Ste:CG33246	protein kinase regulator activity
Gα49B	G-protein coupled receptor binding
Prosα1	threonine-type endopeptidase activity
snRNA:U2:14B	..
RPS28a	structural constituent of ribosome
hkb	sequence-specific DNA binding transcription factor activity
Cht3	chitin binding
Gα49B	G-protein coupled receptor binding
Gα49B	G-protein coupled receptor binding
CG9517	..
dib	iron ion binding
tll	sequence-specific DNA binding transcription factor activity
Gα49B	G-protein coupled receptor binding
skpE	ubiquitin-protein ligase activity
Rae1	protein binding
Odc2	ornithine decarboxylase activity
Prx2540-1	peroxidase activity
Ugt86De	glucuronosyltransferase activity
Adgf-D	adenosine deaminase activity
mre11	endonuclease activity
CCKLR-17D3	neuropeptide receptor activity
CCKLR-17D3	neuropeptide receptor activity
CCKLR-17D3	neuropeptide receptor activity
CCKLR-17D3	neuropeptide receptor activity
λTry	serine-type endopeptidase activity
γTry	serine-type endopeptidase activity

CAH1	carbonate dehydratase activity
Gadd45	..
Atg13	protein kinase regulator activity
aop	sequence-specific DNA binding transcription factor activity
Elo68α	fatty acid elongase activity
CAH1	carbonate dehydratase activity
Vha16-3	proton-transporting ATPase activity, rotational mechanism
Adh	alcohol dehydrogenase (NAD) activity
PH4αSG2	procollagen-proline 4-dioxygenase activity
PH4αSG2	procollagen-proline 4-dioxygenase activity
Elo68β	fatty acid elongase activity
os	receptor binding
Aph-4	alkaline phosphatase activity
upd2	positive regulation of JAK-STAT cascade
Ect3	beta-galactosidase activity
vanin-like	pantetheine hydrolase activity
Gα49B	G-protein coupled receptor binding
cln3	..
Amyrel	cation binding
Sk1	D-erythro-sphingosine kinase activity
WntD	G-protein coupled receptor binding
Ugt86Di	glucuronosyltransferase activity
tll	sequence-specific DNA binding transcription factor activity
Takl2	protein kinase activity
ηTry	serine-type endopeptidase activity
Amy-d	cation binding
Amy-p	cation binding
Gpdh	glycerol-3-phosphate dehydrogenase [NAD+] activity
Gpdh	glycerol-3-phosphate dehydrogenase [NAD+] activity
Gpdh	glycerol-3-phosphate dehydrogenase [NAD+] activity
εTry	serine-type endopeptidase activity
Fad-2	stearoyl-CoA 9-desaturase activity
Gα49B	G-protein coupled receptor binding
Gα49B	G-protein coupled receptor binding
Menl-1	malate dehydrogenase (decarboxylating) (NAD+) activity
Gα49B	G-protein coupled receptor binding
Gα49B	G-protein coupled receptor binding
Wnt10	G-protein coupled receptor binding
hkb	sequence-specific DNA binding transcription factor activity
Mdr50	drug transmembrane transporter activity
cathD	aspartic-type endopeptidase activity
Sodh-2	L-iditol 2-dehydrogenase activity
Hsp70Aa	..
PH4αPV	procollagen-proline 4-dioxygenase activity
Elo68β	fatty acid elongase activity
vanin-like	pantetheine hydrolase activity
b	pyridoxal phosphate binding
εTry	serine-type endopeptidase activity
vri	sequence-specific DNA binding
cln3	..
Ugt37a1	glucuronosyltransferase activity
jhamt	juvenile hormone acid methyltransferase activity
Elo68α	fatty acid elongase activity
Gα49B	G-protein coupled receptor binding
ηTry	serine-type endopeptidase activity
θTry	serine-type endopeptidase activity
Gα49B	G-protein coupled receptor binding
cln3	..
iTry	serine-type endopeptidase activity
αTry	serine-type endopeptidase activity
Dat	arylamine N-acetyltransferase activity
Dat	arylamine N-acetyltransferase activity
vri	sequence-specific DNA binding
b	pyridoxal phosphate binding
aop	sequence-specific DNA binding transcription factor activity
aop	sequence-specific DNA binding transcription factor activity
CAH1	carbonate dehydratase activity
Ugt86Dd	glucuronosyltransferase activity
ζTry	serine-type endopeptidase activity
Ugt86Dg	glucuronosyltransferase activity
Ugt86Dd	glucuronosyltransferase activity
Cht8	chitin binding
Tsp42Eb	..
Uro	urate oxidase activity
Cht4	chitin binding
Thor	eukaryotic initiation factor 4E binding

kkv	chitin synthase activity
Cht3	chitin binding
PH4αSG1	procollagen-proline 4-dioxygenase activity
Gα49B	G-protein coupled receptor binding
Ugt86Dg	glucuronosyltransferase activity
RpS28a	structural constituent of ribosome
Prosα1	threonine-type endopeptidase activity
Gyc32E	guanylate cyclase activity
Hsp70Ab	..
skpE	ubiquitin-protein ligase activity
Vha16-3	proton-transporting ATPase activity, rotational mechanism
Gyc-89Da	guanylate cyclase activity
Rpt3R	ATP binding
CG5389	proton-transporting ATP synthase activity, rotational mechanism
Menl-1	malate dehydrogenase (decarboxylating) (NAD+) activity
Hsp60C	ATP binding
PH4αNE2	procollagen-proline 4-dioxygenase activity
skpD	ubiquitin-protein ligase activity
α4GT2	..
Odc2	ornithine decarboxylase activity
skpC	ubiquitin-protein ligase activity
CG31742	cellular response to DNA damage stimulus
PpD5	protein serine/threonine phosphatase activity
elF4E-3	RNA 7-methylguanosine cap binding
Pros28.1A	threonine-type endopeptidase activity
Prosα6T	threonine-type endopeptidase activity
Dhc98D	microtubule motor activity
Prosβ4R1	threonine-type endopeptidase activity
kkv	chitin synthase activity
kkv	chitin synthase activity
Menl-1	malate dehydrogenase (decarboxylating) (NAD+) activity
Ste:CG33246	protein kinase regulator activity
shf	protein binding
spo	iron ion binding
Hsp70Bp	..
Gα49B	G-protein coupled receptor binding
elF4E-6	RNA 7-methylguanosine cap binding
Gyc32E	guanylate cyclase activity
Gyc32E	guanylate cyclase activity
snRNA:U2:34ABa	..
Gα49B	G-protein coupled receptor binding
Dhc98D	microtubule motor activity
Gα49B	G-protein coupled receptor binding
elF4E-6	RNA 7-methylguanosine cap binding

trpl	cation channel activity
Hsp70Bc	..
Vkor	vitamin-K-epoxide reductase (warfarin-sensitive) activity
βTry	serine-type endopeptidase activity
Rpt3R	ATP binding
Menl-1	malate dehydrogenase (decarboxylating) (NAD+) activity
Amyrel	cation binding
Menl-1	malate dehydrogenase (decarboxylating) (NAD+) activity
elF4E-6	RNA 7-methylguanosine cap binding
Gyc32E	guanylate cyclase activity
CG9517	..
wmd	receptor binding
loopin-1	aminopeptidase activity
Hsp60C	ATP binding
TfIIA-S-2	..
os	receptor binding
Prosβ2R2	threonine-type endopeptidase activity
Trf4-2	DNA-directed DNA polymerase activity
Menl-1	malate dehydrogenase (decarboxylating) (NAD+) activity
Prosα3T	threonine-type endopeptidase activity
Vha16-2	proton-transporting ATPase activity, rotational mechanism
Ssl2	strictosidine synthase activity
RpS10a	structural constituent of ribosome
RpL37b	structural constituent of ribosome
Trf4-2	DNA-directed DNA polymerase activity
gskt	protein serine/threonine kinase activity
Atg8b	protein tag
RpL10Aa	structural constituent of ribosome
Pp1-13C	protein serine/threonine phosphatase activity
Adgf-B	adenosine deaminase activity
nxf4	..
skpF	ubiquitin-protein ligase activity
nmdyn-D7	nucleoside diphosphate kinase activity
CkIIβ2	protein kinase activity
ran-like	GTPase activity
Vha100-3	proton-transporting ATPase activity, rotational mechanism
Prosβ5R	threonine-type endopeptidase activity
elF4E-4	RNA 7-methylguanosine cap binding
PpN58A	protein serine/threonine phosphatase activity
Ssl	strictosidine synthase activity
Pglym87	phosphoglycerate mutase activity
Adgf-E	adenosine deaminase activity
sinah	ubiquitin-protein ligase activity
Cht12	chitin binding

loopin-1	aminopeptidase activity	
Hsc70-2	unfolded protein binding	
Rpn12R	..	
Ubc84D	ubiquitin-protein ligase activity	
Sdic3	microtubule motor activity	
Trxr-2	flavin adenine dinucleotide binding	
Acyp	acylphosphatase activity	
Pros28.1B	threonine-type endopeptidase activity	
Prosβ2R1	threonine-type endopeptidase activity	
Sdic1	microtubule motor activity	
Hsp60C	ATP binding	
