# Supplementary material for: Gene Expression Associated with Early and Late Chronotypes in Drosophila melanogaster
Source: Front Neurol. 2015 May 8;6:100. doi: 10.3389/fneur.2015.00100 (PMC4457141; doi:10.3389/fneur.2015.00100)
Supplement: Supplementary file 5 [file Table_2.PDF]

Supplementary Material

Gene Expression Associated with Early and Late Chronotypes in *Drosophila melanogaster*

Pegoraro M, Picot E*, Hansen C*, Kyriacou CP, Rosato E, Tauber E[$]

Dept. Genetics, University of Leicester, Leicester United Kingdom

***** Equal contribution

[$] **Correspondence:** Dr Eran Tauber Dept. of Genetics. University of Leicester. Leicester LE1 7RH United Kingdom
et22@le.ac.uk

TableS2. Pathway analysis (time-series).

Exp	PathwayID	PathwayName	pORA	pORA_FDR	pSPIA	pG	pG_FDR	Status
DD	dme00982	Drug metabolism - cytochrome P450 -	0	0	.	.	.	.
DD	dme00980	Metabolism of xenobiotics by cytochrome P450	0	0	.	.	.	.
DD	dme00500	Starch and sucrose metabolism	0	0	.	.	.	.
DD	dme00830	Retinol metabolism	0	0	.	.	.	.
DD	dme00040	Pentose and glucuronate interconversions	0	0	.	.	.	.
DD	dme00053	Ascorbate and aldarate metabolism	0	0	.	.	.	.
DD	dme04080	Neuroactive ligand-receptor interaction -	0	0	0.944	0	0	
DD	dme00860	Porphyrin and chlorophyll metabolism	0	0	.	.	.	.
DD	dme00983	Drug metabolism - other enzymes -	0	0	.	.	.	.
DD	dme00232	Caffeine metabolism	0	0	.	.	.	.
DD	dme00480	Glutathione metabolism	0	0	.	.	.	.
DD	dme04310	Wnt signaling pathway	0	0.002	0.018	0	0	
DD	dme03050	Proteasome	0	0	.	.	.	.
DD	dme00062	Fatty acid elongation	0	0.002	.	.	.	.
DD	dme01040	Biosynthesis of unsaturated fatty acids	0	0.003	.	.	.	.
DD	dme00981	Insect hormone biosynthesis	0	0.004	.	.	.	.
DD	dme04013	MAPK signaling pathway	0.002	0.016	0.892	0.014	0.016	
DD	dme00330	Arginine and proline metabolism	0.003	0.018	.	.	.	.
DD	dme04340	Hedgehog signaling pathway	0.01	0.066	0.365	0.024	0.028	
LD	dme04080	Neuroactive ligand-receptor interaction -	0	0	0.044	0	0	
LD	dme00982	Drug metabolism - cytochrome P450 -	0	0	.	.	.	.
LD	dme00980	Metabolism of xenobiotics by cytochrome P450	0	0	.	.	.	.
LD	dme00500	Starch and sucrose metabolism	0	0	.	.	.	.
LD	dme00040	Pentose and glucuronate interconversions	0	0	.	.	.	.
LD	dme00053	Ascorbate and aldarate metabolism	0	0	.	.	.	.
LD	dme00830	Retinol metabolism	0	0	.	.	.	.
LD	dme00860	Porphyrin and chlorophyll metabolism	0	0	.	.	.	.
LD	dme00983	Drug metabolism - other enzymes -	0	0	.	.	.	.
LD	dme00062	Fatty acid elongation	0	0	.	.	.	.
LD	dme00480	Glutathione metabolism	0	0	.	.	.	.
LD	dme00232	Caffeine metabolism	0	0	.	.	.	.
LD	dme00981	Insect hormone biosynthesis	0	0	.	.	.	.
LD	dme00780	Biotin metabolism	0	0	.	.	.	.
LD	dme01040	Biosynthesis of unsaturated fatty acids	0	0.001	.	.	.	.
LD	dme04310	Wnt signaling pathway	0.249	0.742	0.006	0.011	0.013	
LD	dme02010	ABC transporters	0.003	0.026	.	.	.	.

LD	dme00330	Arginine and proline metabolism	0.003	0.026	.	.	.	.
LD	dme04013	MAPK signaling pathway	0.008	0.056	0.436	0.022	0.026	
